# High-Dose Colchicine: Key Factor in the Treatment of Morbidly Obese COVID-19 Patients

**DOI:** 10.7759/cureus.58164

**Published:** 2024-04-13

**Authors:** Tsanko Mondeshki, Vanyo Mitev

**Affiliations:** 1 Propaedeutics of Internal Diseases, Medical University of Sofia, Hospital Alexandrovska, Sofia, BGR; 2 Chemistry and Biochemistry, Medical University of Sofia, Sofia, BGR

**Keywords:** cytokine storm, nlrp3 inflammasome, covid-19, comorbid obesity, colchicine

## Abstract

Colchicine has long been known to possess anti-inflammatory effects by inhibiting microtubules, activation and migration of neutrophils, and most importantly, the inflammasome complex found in neutrophils and monocytes. Due to these properties, a number of clinical trials have tested the therapeutic effect of colchicine in COVID-19 patients.

One common feature of these studies, however, is the low therapeutic dose used, which may explain the conflicting and disappointing results. Colchicine has the unique property of accumulating in leukocytes, which are primarily responsible for the hyperactivation of the NLRP3 inflammasome and the cytokine storm. The low-dose colchicine used to treat COVID-19 is not sufficient to reach the necessary intracellular concentration for NLRP3 inflammasome inhibition. We have reported our experience with high-dose colchicine, within the approved therapeutic range, in both ambulatory and hospitalized patients, and have shown dramatic cure rates.

Here, we present our observation of an excellent therapeutic effect of high-dose colchicine in morbidly obese COVID-19 patients who are at the highest morbidity and mortality risk.

## Introduction

Obesity is an independent risk factor for the development of respiratory failure and mortality during virus pandemics, including COVID-19 [1]. It is also well known that obesity is associated with significantly increased rates of hospitalization, mechanical ventilation, and higher mortality among COVID-19 patients [2-4]. Obesity is among the major factors in endothelial dysfunction, the key pathophysiologic event leading to increased mortality in COVID-19 [5,6].

Obesity affects approximately 13% of the world’s population, becoming one of the most prevalent and socially significant conditions [7]. It is now well established that obesity-related comorbidities such as hypertension, cardiovascular disease, type 2 diabetes, and altered cortisol metabolism put overweight patients with COVID-19 at an increased risk of life-threatening complications [8].

We have previously demonstrated that using high-dose colchicine in the therapeutic regimen of severely ill hospitalized COVID-19 patients leads to a significantly increased recovery rate and decreased mortality rate between two and seven-fold, depending on the dosage [9-12].

Very recently, we described a case of a patient with severe obesity and multiple comorbidities who, despite standard hospital treatment, deteriorated sharply, but his life was saved as a result of the included treatment with high doses of colchicine [13].

Here, we present a case series of three morbidly obese patients with multiple comorbidities and severe COVID-19, who rapidly recovered and were cured with our unique therapeutic regimen, the hallmark of which is the high dose of colchicine. The first two cases were high-risk COVID-19 outpatients. Our therapeutic regimen has been reviewed, approved, and widely accepted for the treatment of COVID-19 patients by the Interdepartmental Concilium of University Hospital “Alexandrovska.” The colchicine dose used for the treatment of COVID-19 patients was within the therapeutic range of the medication, and it is calculated by the formula: loading dose [0.5 mg/10 kg body weight] - 0.5 mg (up to and not exceeding 5 mg), followed by a maintenance daily dose that is half of the loading dose [9,10].

The severe complications of COVID-19 are known to be related to hyperactivation of NLRP3 inflammasome - the main target of our high-dose colchicine treatment regimen. Although the COVID-19 pandemic is almost over, we believe the inhibition of NLRP3 inflammasome with high doses of colchicine to be a valuable treatment approach for other infectious conditions related to abrogated NLRP3 response.

## Case presentation

Case 1

A 34-year-old morbidly obese man, active cigarette smoker, with a BMI of 52.6 kg/m^2^ and insulin resistance, presented to his family doctor with a fever of 38.5°С, oxygen saturation of 90%, and malaise. His blood workup, including a complete blood count (CBC) and a comprehensive metabolic panel (CMP), was unremarkable, except for C-reactive protein (CRP) of 23.5 mg/l (0-5 mg/l) and D-dimer of 675.0 ng/ml (0-500 ng/ml). A computed tomography (CT) of the chest showed ground-glass opacities in bilateral apical lung fields. He was diagnosed with COVID-19 by a polymerase chain reaction (PCR) test. He was prescribed betamethasone i.m. (1 amp. 7 mg/ml) every other day and vitamin C. The next seven days, his general condition got progressively worse with fevers (up to 39°C), muscle pain, and decreasing oxygen saturation (85-88%). He was referred to and consulted by our group via a tele-visit consultation and started on the following therapeutic regimen: colchicine 0.5 mg tablets by mouth with a loading dose of 5 mg for the first 24 hours, followed by a maintenance dose of 1 mg every eight hours; bromhexine hydrochloride (BHH) nebulizer inhalations 1 ampoule diluted in 2 ml of 0.9% NaCI three times a day (TID). The following day, the patient was afebrile, the oxygen saturation increased to 92%, and the general condition improved significantly. Normal oxygen saturation was achieved on day seven after initiation of colchicine therapy. The fast recovery of COVID-19 patients is a hallmark of our high-dose colchicine treatment. Maintenance therapy with colchicine 2.5 mg continued for 10 days. During the entire treatment period, the patient reported no diarrhea or other gastrointestinal (GI) symptoms. The outpatient avoided hospital admission, therefore no following X-ray is available.

Case 2

А 50-year-old morbidly obese man, with a BMI of 61.3 kg/m^2^, hypertension, obstructive sleep apnea, and chronic venous insufficiency of lower extremities presented to his family physician with a fever of 39.2°C, dry cough, general fatigue, and muscle pain. He was diagnosed with COVID-19 by a rapid nasopharyngeal antigen test. His social history was significant for smoking cigarettes 40 packs/year for 20 years.

Except for elevated CRP of 52 mg/l (0-5 mg/l), his blood workup, including a CBC and a CMP, was unremarkable. He was started by his family doctor on paracetamol 500 mg p.o. four times a day and ibuprofen 400 mg p.o. four times a day. Over the course of the following five days, his overall status and complaints got significantly worse with shortness of breath during usual physical activity, and high-grade fevers of 38.5°C. After a tele-visit consultation with our group, the patient began treatment with the following: nebulizer inhalations of BHH 1 ampoule diluted in 2 ml of normal saline, three times a day; colchicine tablets of 0.5 mg each (5 mg loading dose for the first 24 hours, followed by a maintenance dose of 1 mg every eight hours); famotidine 40 mg p.o. three times a day. In 48 hours, the patient was afebrile and reported a significant improvement in his general condition without shortness of breath or muscle pain. The fast recovery of the outpatient is worth pointing out again. The therapy continued for 10 days, after which the dose of colchicine was reduced to three tablets per day distributed at eight-hour intervals for 30 days. During the entire treatment period, the patient reported no GI symptoms. The outpatient avoided hospital admission, therefore no following X-ray is available.

Case 3

A 53-year-old morbidly obese man with a BMI of 50.8 was admitted to the hospital for the treatment of COVID-19-associated bilateral pneumonia and respiratory failure. His past medical history was significant for poorly controlled hypertension, type 2 diabetes mellitus on insulin replacement, and obstructive sleep apnea on bilevel positive airway pressure (BPAP).

The patient's initial symptoms began eight days prior to hospitalization. The patient reported an elevated temperature of up to 38.5°C, dry ineffective cough, chest pressure, and shortness of breath with minimal physical exertion. Treatment with paracetamol 500 mg TID and nimesulide 100 mg twice a day (BID) was started. However, the effect on his overall condition was limited and unsatisfactory, and he remained subfebrile up to 37.4°C. The patient visited his family doctor two days later, who started him on oral antibiotic azithromycin 500 mg for five days. His rapid nasopharyngeal antigen test for COVID-19 was positive. In the following days, the patient reported a significant decline in his general condition with worsening cough, fevers, and shortness of breath. A follow-up visit to his family practitioner revealed that his oxygen saturation was 87% at rest with a heart rate of 115 beats per minute and an arterial blood pressure of 105/75. Because of that, he was referred to the ER and was admitted to the hospital for further management.

**Figure 1:**
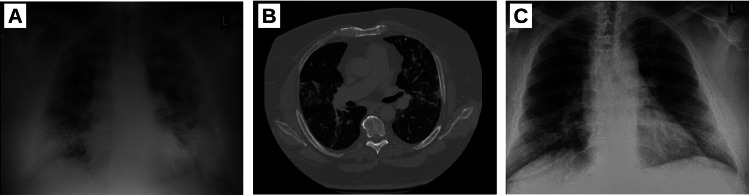
Radiological imaging (A) Chest X-ray prior to hospital admission with bilateral infiltrative lung changes. (B) Representative axial image of chest CT scan with scattered ground-glass opacities. (C) Follow-up chest X-ray showing resolution of the bilateral infiltrative lung changes.

Upon admission to the infectious disease unit of the hospital, a chest X-ray showed bilateral opacities (Figure 1A). The CT scan of the chest showed bilateral scattered small to medium foci of “crazy paving” in all segments bilaterally, as well as areas of ground glass, with no areas of consolidation (Figure 1B). Bilateral small modules were observed in all segments without pleural effusions and without pericardial effusion. There were enlarged subcarinal lymph nodes.

The patient's therapeutic regimen included the following: oxygen 10 l/min delivered by nasal cannula; nebulizer inhalations with BHH TID; colchicine 0.5 mg tablets 5 mg (first-day loading dose), followed by a maintenance dose of 3 mg daily (two tablets every eight hours); methylprednisolone 120 mg i.v. TID for three days, followed by 40 mg TID; famotidine 40 mg i.v. BID; oral hymecromone 400 mg tablet, two tablet BID; antibiotic meropenem 2g i.v.; heparin i.v. bolus of 10,000 U, followed by 2000 U/hour. He was also started on an insulin drip.

The patient’s general condition improved very quickly and, on hospital day two, the systemic inflammatory response syndrome was under control. He remained afebrile for the remainder of the hospital stay with improving inflammatory markers (Table 1). On day four after the initiation of colchicine intake, the patient started having diarrhea (five times/day). Thus, the colchicine dose was reduced to 0.5 mg TID.

**Table 1:** Laboratory tests of patient 3 Laboratory tests of patient 3 during the hospital stay. PCR: polymerase chain reaction; MCV: mean corpuscular volume; MCH: mean corpuscular hemoglobin; MCHC: mean corpuscular hemoglobin concentration; MPV: mean platelet volume; PDW: platelet distribution width; pCO_^2^_: partial pressure of carbon dioxide; pO2: partial pressure of oxygen; tCO2: total carbon dioxide; CRP: C-reactive protein; BE: base excess; SB: standard bicarbonate.

Laboratory test		Normal range		11.12.2021, 19:00	14.12.2021, 21:40	17.12.2021, 12:30	20.12.2021, 08:20	26.12.2021, 08:50
PCR test COVID-19 (SARS-CoV-2 RNA)				(+) Positive				(-) Negative
Leukocytes (Leu)	10^9/l	3.5	10.5	5.0	5.7	6.7	6.4	10.6
Erythrocytes (Er)	10^12/l	4.4	5.9	5.5	5.5	5.8	6.1	6.0
Hemoglobin (Hb)	g/L	135	180	158	154	164	172	172
Hematocrit (Ht)	g/L	0.4	0.53	0.49	0.48	0.52	0.55	0.54
MCV	fL	82	96	89	87	90	90	90
MCH	pg	27	33	29	28	29	28	29
MCHC	g/L	300	360	323	324	317	316	318
Platelets (Tr)	10^9/l	130	440	134	117	159	208	183
MPV	fL	6.1	11.5	11.3	11.3	11.8	11.5	12.0
PDW	%	8	23	14.1	15.6	15.4	14.5	15.0
NE % - Neutrophil granulocytes %	%	40	70	53	81	91	86	89
EO % - Eosinophilic granulocytes %	%	0	6.5	1.0	0.0	0.0	0.2	0.0
BA% - Basophilic granulocytes %	%	0	2	1	0	0	0	0
MO % - Monocytes %	%	1	11	16.8	5.1	2.8	6.6	5.2
LY % - Lymphocytes %	%	20	48	29	14	6	7	5
NE# - Neutrophil granulocytes count	10^9/l	2	7	2.6	4.6	6.1	5.5	9.5
ЕО# - Eosinophilic granulocytes count	10^9/l	0	0.5	0.1	0.0	0.0	0.0	0.0
BA# - Basophilic granulocytes count	10^9/l	0	0.14	0.03	0.01	0.00	0.01	0.02
MO# - Monocytes count	10^9/l	0	0.8	0.8	0.3	0.2	0.4	0.6
LY# - Lymphocytes count	10^9/l	1	4	1.4	0.8	0.4	0.5	0.6
IG (%) - Immature granulocytes - %	%	0	5	0.4	0.4	0.1	0.8	1.3
IG # - Immature granulocytes - count	10^9/l	0	0.7	0.0	0.0	0.0	0.1	0.1
D-dimer	µg/l	0	0.55	1.2	0.94	0.22	0.43	0.26
Creatinine - serum	µmol/l	62	106	107	121	99	76	82
pH		7.35	7.45	7.4	7.45			7.39
pCO_2_	kPa	4.67	6	3.5	4.11			4.5
pO_2_	kPa	10	13	6.75	7.55			9.2
SB	mmol/l	21	25	20.5	23.7			24.2
BE (w)	mmol/L	-2.5	2.5	-3.3	-2.2			2.1
O_2_ Sat	%	94	98	88	91			93
tCO_2_	mmol/l	20	27	19.5	21.5			22.5
CRP	mg/l	0	5	148	148.1	36.7	11.1	1.0
Ferritin	ng/ml	30	400	1520	1305	1021	943.0	829.3
Procalcitonin 1	ng/ml					0.151	0.091	0.073

The patient’s condition continued to improve and he was discharged on day 15 of hospitalization independent of supplemental oxygen and with a negative PCR test for COVID-19. The therapeutic regimen on discharge included a maintenance dose of colchicine 0.5 mg TID for 30 days and apixaban 5 mg tablets BID.

At a two-week follow-up, the patient continued to feel well without subjective complaints. Laboratory parameters were within normal limits, and chest X-ray showed resolution of the bilateral infiltrative changes (Figure 1C).

## Discussion

We present three high-risk morbidly obese patients with severe COVID-19, two of whom did not require hospital admission after a timely and successful treatment with high doses of colchicine. The third patient was initially treated without colchicine by his primary care physician and eventually required admission to the hospital on day eight of the onset of COVID-19. The inpatient therapy with high doses of colchicine has led to a quick recovery and saved the patient's life.

We have shown in the past that our treatment regimen, including BHH nebulizers, higher doses of colchicine, and hymecromone, has a tremendous effect on hospitalized patients with COVID-19 [9,10].

The effectiveness of BHH is maximized when given prophylactically to high-risk patients by inhalation, after contact with a COVID-19 patient or carrier. In cases in which the COVID-19 diagnosis has been already established, BHH inhalations are helpful in limiting the infection; however, it is not as effective, because the virus has already penetrated. Hymecromone is given to inpatients to prevent a possible hyaluronan storm [9-11]. The most important therapeutic focus in patients with COVID-19, however, is the blockage of the NLRP3 inflammasome formation [12]. Therefore, colchicine can be successfully used to prevent the worsening of COVID-19 symptoms in ambulatory conditions as well as for treatment of severe cases requiring hospitalization [12].

The WHO-recommended strategy to inhibit viral replication (Paxlovid, remdesivir, and molnupiravir) has been partially successful because there is no direct link between viral load and hyperactivation of the NLRP3 inflammasome [12,14]. On the other hand, these medications have serious side and mutagenic effects [15-17].

Inhibiting just interleukin-6 (IL-6), one of a myriad of elevated cytokines associated with the formation and overactivation of the NLRP3 inflammasome, is not the best strategy. Another strategy using baricitinib to inhibit the Janus kinase/signal transducer and activator of transcription (JAK/STAT) pathway, which is activated by and downstream to many cytokines, seems more promising [12]. However, inhibiting the formation and activation of NLRP3 inflammasome with our economically affordable and favorable therapeutic approach makes the use of extremely expensive drugs, such as baricitinib, meaningless [12].

These cases support our previously published data and highlight two important take-home messages: (1) if given on time to patients susceptible to severe COVID-19, high-dose colchicine prevents hospitalization and helps with recovery without complications; (2) high-dose colchicine, as part of our therapeutic strategy, is associated with significantly improved prognosis and significantly decreased mortality in hospitalized critical ill COVID-19 patients.

We have discussed the rationale of our successful treatment approach in greater detail in our previous publications [12]. In brief, successful treatment of COVID-19 is only possible if the hyperactivated NLRP3 inflammasome is inhibited on time. To the best of our knowledge, the only possible way to accomplish this is with higher doses of colchicine, due to its remarkable property to be actively accumulated in the white blood cells where the COVID-19 cytokine storm is generated [9-11].

A large number of viruses can activate the NLRP3 inflammasome [18]. Annually, after each flu, there are deaths whose pathogenetic mechanism goes through an overactivation of the NLRP3 inflammasome [19]. We are convinced that colchicine would be useful in these cases as well. Future studies need to be conducted to test for this hypothesis and we have already started preliminary clinical tests.

High-dose colchicine can be used safely to treat COVID-19, if patients do not have liver or kidney insufficiencies, and adverse drug interactions are avoided [20].

## Conclusions

These three cases clearly demonstrated the life-saving effect of high-dose colchicine. Taking into consideration the fact that obese patients with COVID-19 have an increased risk of life-threatening complications, we recommend the application of high colchicine doses as a mandatory therapeutic scheme.
